# Thalamotomy for essential tremor: FDA approval brings brain treatment with FUS to the clinic

**DOI:** 10.1186/s40349-017-0096-9

**Published:** 2017-07-13

**Authors:** Paul S. Fishman

**Affiliations:** Department of Neurology, University of Maryland School of Medicine, Baltimore, MD 21201 USA

In July of 2016, the FDA granted approval for MR guided focused ultrasound (MRgFUS) mediated unilateral lesioning of the ventral intermediate nucleus (VIM) of the thalamus for treatment of medically refractory and disabling essential tremor (ET). The approval was the culmination of decades of progress in scientific and clinical research on the application of FUS to the brain. This progress includes improved technology to provide controlled levels of ultrasonic energy that can be focused to a brain target non-invasively through the skull, and guided by MR imaging. High intensity focused ultrasound (HIFU) now can create a coagulation based stereotactic brain lesion in a substantially less invasive manner than an open surgical procedure. In spite of this progress, there is only the beginning of awareness of ultrasound as a therapeutic modality for neurologic disease. With this recent and first FDA approval the time is here to widen the circle with understanding of the potential applications of MRgFUS for treatment of the brain.

ET became the first neurological disorder targeted for treatment with modern MRgFUS for clear reasons. ET is a very common disorder where medical therapy is frequently inadequate for patients with severe and disabling tremor [1, 2]. There is extensive experience showing reduction of tremor in medically refractory patients with ET though targeting of the VIM of the thalamus with either stereotactic lesioning or deep brain stimulation (DBS). The VIM is centrally located within the brain, reducing the impact of the skull in absorbing and distorting focused sonic energy, the major historical impediment in the clinical application of this technology, and is within the treatment envelope of the current devices. In just the past few years independent published clinical studies have shown significant improvement after treatment in both tremor amplitude and tremor related disability with unilateral MRgFUS mediated thalamotomy [3–7]. These studies were quickly followed by a pivotal double-blind, sham-controlled, multicenter study that resulted in FDA approval [8]. The decrease seen in arm tremor with treatment in these studies has been consistently not only statistically significant, but also showing robust reduction in both tremor amplitude and related disability. The magnitude of improvement in these medically refractory patients clearly supports MRgFUS as a new treatment option for patients with ET.

The publication of the pivotal study immediately generated interest in the question of how this new treatment option compares to the current surgical standard of care for medically refractory and disabling ET—deep brain stimulation (DBS) [9]. The general strategy of both surgical treatments for ET is the same: To attain maximal reduction of tremor without unwanted neurologic symptoms related to effects on brain tissue surrounding the target region.

Although there are no comparison trials at this time, the magnitude of reduction in hand tremor in the pivotal study (approximately 40%) is at the bottom of the cited range for unilateral VIM DBS for ET (40–85%, [10]). MRgFUS mediated thalamotomy appears to be more comparable to unilateral thalamic DBS when evaluating tremor related disability (approximately 60% reduction with FUS, 40–80% reduction range for DBS, [11]). The wide cited range for DBS benefit reflects a large number of studies with varying designs and outcome measures, but does not include a study with a sham-controlled design used in the FUS/ET pivotal trial.

The side effect profiles of the two treatment strategies are also very different. Physicians familiar with DBS are quick to note that MRgFUS unlike DBS has the goal of making a permanent brain lesion. Some FUS studies have frequent adverse effects of treatment, where numbness or paresthesia is the most common neurologic symptom (38% transient, 14% at one year in the pivotal study). Serious side effects of FUS mediated thalamotomy however have been rare at this point. Only one out of fifty treated patients in the pivotal trial reported a dense hypoethesia of the hand that was rated as the only severe adverse event. Neurologic symptoms after MRgFUS are likely related to spread of the sonic/thermal lesion to brain regions neighboring the target. The pivotal study of MRgFUS included several centers (including our own) with no previous patient experience with this technology. It has been suggested (as is the case for many therapeutic procedures) that more experienced centers have better outcomes [12]. Although plausible hypothesis, it remains to be demonstrated if increased experience with the MRgFUS patient procedure will improve the ratio of durable benefit to off target symptoms.

DBS has the well-recognized serious complications of any open neurosurgical procedure of intra-cerebral hemorrhage (0.5%–2.0%) and infection (1–3%). There are also as DBS-specific issues such as lead migration and fracture (1–3%), and device malfunction which can frequently require surgical correction (1–3%) [13–15]. DBS also requires a second surgical procedure to implant the programmable impulse generator in the chest, which needs periodic surgical replacement. These limitations of DBS have played a role in limiting its patient acceptance, as well as provided a rationale for investigating less invasive surgical methods for the treatment of movement disorders [16].

Serious intracranial hemorrhage was reported in the first attempt to use MRgFUS in the brain to ablate malignant brain tumors [17]. Since that experience, current approaches rely on MRI to avoid brain regions with abnormal vasculature. At this point with over 400 patients treated with MRgFUS for functional brain ablation worldwide, there have been no reports of intracerebral bleeding or infection with this procedure that does not open the skull or meninges.

Unlike DBS, there is little potential and no current evidence of brain damage along the path to the target. Using the converging multi-emitter approach of current MRgFUS devices, brain temperatures only a few millimeters away from the planned target rarely rise significantly above normal. This is in contrast to DBS when the rigid cylinders of electrodes and cannulas are passed through centimeters of non–target tissue, with the potential for unintended brain injury.

The patient experience for DBS and MRgFUS has both similarities and substantial differences. Both procedures localize the target with MRI and plan their approach using a stereotactic frame firmly fixed to the patients shaved head. Microelectrode recording commonly used for target validation in DBS is not possible with the close skull, incision-less approach of MRgFUS, which instead uses MR thermography for real time measurement of target brain temperature to control lesion size and location. During both DBS and MRgFUS, the intensity of electrical stimulation or sonic/thermal energy is gradually increased, and the response of the awake patient (off medication for their movement disorder on that day to maximize these symptoms) with relief of motor symptoms or intrusion of other neurologic symptoms to functionally validate the accuracy of the anatomic target and determine the treatment endpoint.

At this point MRgFUS mediated thalamotomy has only been performed unilaterally, restricting benefit to one body side. Part of the rationale for the development of DBS was earlier experience with lesion based tremor surgery, where side effects such as worsening of speech commonly occurred with bilateral thalamic lesions [18]. DBS is commonly targeted to the VIM on both sides, with improvement of both bilateral limb tremor as well as axial tremor. With the adjustable DBS systems, intrusion of these unwanted neurological symptoms can frequently be reduced by changes in stimulation parameters [19]. A recent meta-analysis of the speech related complications of both thalamotomy and DBS, suggests that the long-standing view of this risk of thalamotomy may be inaccurate [20]. Although the overall patient risk of worsening speech was high for both unilateral (15%) and bilateral (40%) thalamotomy, the risk among ET patients was much lower (4.5% for unilateral and 13% for bilateral lesions). The rate of speech worsening in the ET population after thalamotomy was found to be lower than with DBS. This more contemporary view of risk of speech worsening after thalamotomy is consistent with a recent study of bilateral thalamic lesioning using radio-surgery (Gamma Knife), where only 2 of 68 patients had worsening speech after staged bilateral thalamic lesions for treatment of tremor [21]. Although both MRgFUS and radiosurgery are incisionless stereotactic procedures that have been applied to treatment of tremor, MRgFUS unlike radiosurgery produces a lesion in real time. Although it is possible that the slow developing lesional effect of radiosurgery could have some physiologic advantage, the real time lesioning effect of MRgFUS allows for the avoidance of a permanent thermal lesion in brain regions where speech worsening is seen with a lower energy sonication. It is not surprising that more contemporary thalamic lesioning studies do not observe the severity or incidence of dysarthria seen in early surgical studies. MRgFUS creates a heat/coagulation based thalamic lesion with precise, controllable incisionless technology that bears little resemblance to the original stereotactic surgical thalamotomy introduced during the 1960’s. A clinical safety and efficacy trial of staged bilateral MRgFUS thalamotomy for ET should be seriously considered at this time.

Another immediate question for the use of MRgFUS ablative surgery in the brain is the durability or permanency of the effect of treatment. At this point in time, there is little published experience with long-term follow-up of treated patients with this chronic condition. In the pivotal study, although there is a trend toward an increase in tremor in patients with ET by a year after treatment, the benefit of treatment still remained substantial (47% reduction in tremor score from baseline at 3mos compared to 40% reduction at 12mos). ET and PD are both progressive conditions where motor symptoms worsen over time. The well-established strategy to ameliorate such worsening in patients treated with DBS is through follow-up visits where the parameters of stimulation are adjusted. Unfortunately disease progression may eventually result in worsening symptoms in many patients in spite of this re-programming, particularly in patients with PD [22]. When significant worsening of motor symptoms occurred after open lesional surgery, repeat surgery was occasionally performed, and was usually successful in regaining the original clinical response [23]. Repeat DBS implantation also has a reasonable likelihood of success in patients with poor motor symptom control due to sub-optimal location of the original electrode [24]. This suggests the possibility of benefit in the future of a second attempt at MRgFUS in the patients with less than optimal benefit from the original procedure, as well as retreatment in patients where symptoms re-occur or significantly worsen. The potential efficacy and safety of re-treatment with MRgFUS has not yet been explored, and is a clear target for future clinical research, beginning with ET patients.

Following successes with ET, MRgFUS is rapidly being explored in the treatment of PD, the most common indication for DBS. The VIM has been targeted with FUS for relief of medically refractory and disabling tremor associated with PD in both open label and sham controlled trials [25, 26]. There is initial experience with other targets for non-tremor motor symptoms of PD, with a safety and feasibility trial of MRgFUS mediated unilateral pallidotomy in progress for PD patients with significant dyskinesias [27, 28].

A clinical demonstration of the safety of either bilateral treatment or re-treatment in the initial ET population would have strong implications for future treatment studies of PD with MRgFUS where a large number of patients have both bilateral and progressively worsening symptoms.

Although MRgFUS has been most extensively applied to patients with movement disorders, it has potential for all conditions amenable to stereotactic functional neurosurgery. These include chronic neuropathic pain and psychiatric conditions such as obsessive-compulsive disorder, which have already had promising early clinical trials [6, 29]. The application of MRgFUS to epilepsy remains a technical challenge where affected regions of brain such as cortex and hippocampus are close to the skull. Clinical trials of MRgFUS for ablation of sub-cortical epileptic foci have recently begun, while both newer devices and ablation strategies are under development [17, 30].

Targeted brain ablation as approved for ET, represents only one aspect of the application of MRgFUS to brain disease. Extensive pre-clinical research has developed strategies for using FUS for controlled, targeted and safe opening of the blood–brain barrier (BBB) [31, 32]. This application which combines lower levels of pulsed sonic energy with the use of circulating microbubbles commonly used for diagnostic ultrasound has reached the clinical trial stage for enhancing chemotherapy of malignant brain tumors. MRgFUS mediated opening of the BBB represents the first real advance in this field since the introduction of hyperosmolar mannitol injections in the 1970s, and has direct implications for molecular and cellular therapy of not only brain tumors but neurodegenerative diseases such as Alzheimer’ disease and Parkinson’s disease [33–38]. Applying lower levels of sonic energy to the head can also stimulate both superficial and deep regions of the brain, adding FUS to the toolbox of methods for non-invasive neuromodulation [39].

Over the last decade the number of publications of animal and human studies utilizing therapeutic ultrasound to the brain has increased exponentially. The first FDA approval for treatment of a neurological condition with MRgFUS has clearly enhanced its awareness among a widening community of clinicians and scientists, and will further accelerate the application of this novel, versatile and multi-disciplinary technology.
